# Persistent descending mesocolon (PDM) versus Non-PDM patients on postoperative outcomes after colorectal surgery

**DOI:** 10.1186/s12893-026-03611-x

**Published:** 2026-03-02

**Authors:** Chenyu Xiang, Dong Peng, Junnan Zhao, Lang Wang, Xiaoyu Liu, Guangyan Ji, Ziwei Wang, Donglin Du

**Affiliations:** Department of Gastrointestinal Surgery, The First Affiliated Hospital of Chongqing Medical University, Chongqing, China

**Keywords:** Persistent descending mesocolon, Postoperative outcomes, Colectomy, Colorectal cancer

## Abstract

**Purpose:**

To compare the surgical outcomes between patients with and without Persistent Descending Mesocolon (PDM) undergoing colorectal resection.

**Methods:**

A systematic search of electronic databases (PubMed, Embase, Cochrane Library and Web of Science) was conducted for studies published from inception to October 13, 2025. Statistical heterogeneity was assessed using the I^2^ statistic and the Cochran’s Q test (with a significance level of *P* < 0.1). High heterogeneity was considered when I^2^ > 50%, in which case a random-effects model was applied. For studies with I^2^ ≤ 50%, a fixed-effects model was used, and a *P*-value < 0.05 was considered statistically significant. All analyses were performed using Stata software (version 18.0). The registration ID of this current meta-analysis on PROSPERO is CRD420251166710.

**Results:**

Six observational studies, encompassing a total of 2437 patients (PDM: n=154; Non-PDM: n=2283), were included in the final analysis. Patients in the PDM group had a significantly longer operative time (MD: 28.43minutes, 95% CI: 2.71 to 54.16, p = 0.03). However, no significant difference was found in intraoperative blood loss and postoperative hospital stay between the PDM group and the Non-PDM group. Furthermore, no significance was found in overall complications (OR=1.14, 95% CI = 0.60 to 2.16, P = 0.69).

**Conclusion:**

The presence of PDM is associated with increased surgical complexity, evidenced by longer operative times. Surgeons should be aware of this anatomical variant during preoperative planning for colorectal procedures. No statistically significant differences were observed in postoperative outcomes between PDM and Non-PDM groups.

**Supplementary Information:**

The online version contains supplementary material available at 10.1186/s12893-026-03611-x.

## Introduction

Colorectal cancer (CRC) ranks as the third most commonly diagnosed malignancy and a leading cause of cancer-related deaths worldwide, for which surgical resection remains the cornerstone of curative treatment [1, 2]. Laparoscopic colorectal surgery, including right and left hemicolectomy, anterior resection, and low anterior resection, have become the standard of care for CRC, as well as for a myriad of other conditions such as diverticular disease and inflammatory bowel disease [3, 4]. While surgical techniques and perioperative care have advanced significantly, patient-specific variations remain a critical factor influencing surgical difficulty and postoperative recovery [5–7]. Among these variations, Persistent Descending Mesocolon (PDM) represents a notable embryological anomaly with profound implications for surgical dissection [8–11].

PDM is characterized by the failure of the descending colon’s mesentery to fuse with the posterior parietal peritoneum during fetal development [12, 13]. This results in a mobile and unattached descending colon, a stark contrast to the normal anatomical fixation of the retroperitoneum. From a surgical perspective, this aberrant anatomy poses substantial intraoperative challenges. The absence of the usual avascular plane of Toldt can lead to difficulties in mobilizing the colon, an increased risk of misidentifying anatomical planes, and potential injury to the mesocolic vessels or the underlying duodenum, kidney, and ureter [14, 15]. Consequently, procedures such as left hemicolectomy or anterior resection in patients with PDM are often considered technically demanding [16–19].

To date, there has been no comprehensive synthesis of the available evidence to provide a definitive comparison of surgical outcomes between patients with and without PDM. Therefore, the primary objective of this pooling up analysis is to systematically evaluate the existing evidence to quantify the impact of PDM on intraoperative complications, including operative time, blood loss, and postoperative complications, including anastomotic leak, ileus and overall complications following colorectal surgery. This analysis aims to contribute to improving patient care for this unique anatomical disease.

## Methods

This study was conducted as pooling up analysis of observational comparative studies. The protocol for this analysis was registered prospectively in the International Prospective Register of Systematic Reviews (PROSPERO) with the registration number CRD420251166710. And the link is https://www.crd.york.ac.uk/PROSPERO/view/CRD420251166710 [20].

### Literature search

A comprehensive and systematic literature search was performed, by two authors independently through October 13, 2025, across major electronic databases: PubMed, Embase, Cochrane and Web of Science. The search strategy was performed with the following items (“Persistent descending mesocolon” OR “PDM” OR “Congenital descending mesocolon” OR “Unfused descending mesocolon” OR “ Descending mesocolon anomaly”) AND (““Colorectal Cancer” OR “Colorectal Carcinoma” OR “Colorectal Neoplasm” OR “Colorectal Tumor” OR “Colon Cancer” OR “Rectal Cancer” OR “Colorectal Neoplasms”). This systematic review is focused exclusively on CRC resections. We do not include studies on benign colorectal surgeries. The publication language had no limitations in this search.

### Inclusion and exclusion criteria

The inclusion criteria for this meta-analysis were as follows: (1) Patients with a confirmed diagnosis of PDM, identified either preoperatively by computed tomography (CT) imaging or intraoperatively by surgical findings. (2) Reported quantitative data on outcomes of surgery, such as operative time, intraoperative complications or anastomotic leakage. The exclusion criteria were as follows: (1) Case reports, case series without a comparative control group, review articles, editorials, letters, conference, and comments. (2) Publications with insufficient data that could not be extracted. (3) Studies that did not provide separate outcome data for the PDM and Non-PDM cohorts, or where the full text was unavailable despite efforts to obtain it.

### Study selection

Two authors searched the databases independently. The title and abstract of the articles were screened for relevance first, and the full texts were then evaluated according to the inclusion and exclusion criteria. All disputes were resolved through internal discussion, and if the two authors had disagreed, the third author made a final judgment.

### Data extraction

The data from the included literature were extracted by two authors respectively. The contents extracted were as follows: first author, publication year, study date, country, baseline information. The primary outcome is postoperative complications (as defined by Clavien-Dindo grade II or higher), and the secondary outcomes are operative time, intraoperative complications, anastomotic leak, estimated blood loss, conversion to open surgery, and length of hospital stay.

### Postoperative complications

The assessment of postoperative complications in this meta-analysis was standardized using the Clavien-Dindo classification [21]. In this system, Grade ≥ II complications require pharmacological treatment beyond basic antiemetics or analgesics, while Grade ≥IIIb complications mandate surgical, endoscopic, or radiological intervention under general anesthesia. Furthermore, specific complications pertinent to colorectal surgery were extracted, including anastomotic leakage, intra-abdominal or pelvic infection, postoperative ileus, surgical site infection, bleeding requiring intervention, bowel obstruction.

### Outcomes

The primary outcome of the current meta-analysis was the postoperative complications. Secondary outcomes included intraoperative blood loss, operation time, total number of retrieved lymph nodes, conversion to open surgery, and postoperative hospital stay. Overall complications were defined as any complications, including those classified as Clavien-Dindo grade I (mild) through grade V (death), which encompasses both intraoperative and postoperative complications.

### Quality assessment

The Newcastle-Ottawa Scale (NOS) was used to evaluate the quality of the included studies. The studies were judged from three perspectives: selection of Study comparisons, comparability between groups, and the determination of results [22]. Selection of Study comparisons: This domain examines whether the study participants were representative of the population and whether they were selected in a way that minimizes selection bias. Comparability between groups: This domain assesses whether the study accounted for confounding factors, particularly in observational studies. Determination of results: This domain considers the method of outcome measurement, including whether outcomes were assessed by blinded assessors or whether there was any possibility of outcome reporting bias. Each study was assigned a NOS score reflecting its risk of bias, and studies with lower scores were considered more cautiously in our analysis.

### Statistical analysis

In this article, continuous variables are presented as the mean and standard deviation (SD), while dichotomous variables are presented as proportions. For the pooled analysis of dichotomous and continuous outcomes, odds ratios (ORs) and mean differences (MDs) were calculated, respectively, each with their 95% confidence intervals (CI). We recognized that some studies may report zero events for certain complications. For these cases, we applied a continuity correction to avoid issues with zero-event cells, specifically by adding a small constant (e.g., 0.5) to both the numerator and denominator in the calculation of odds ratios. For studies that reported medians and interquartile ranges (IQRs) instead of means and standard deviations (SDs), we applied the method described by Wan. This approach allows for the estimation of the mean and SD based on the reported median and IQR values. Specifically, we used the method proposed by Hozo, which was refined by Wan, to estimate these parameters: Mean Estimation: The mean was estimated using the following formula: $\:\widehat{X}\approx\:\frac{a+2m+b}{4}$. where a is the minimum value, m is the median, and b is the maximum value. Standard Deviation Estimation: The standard deviation was estimated using the formula: $\:\widehat{S}\approx\:\frac{b-a}{4}$. where b and a are the maximum and minimum values, respectively. This method is based on the assumption that the data are approximately normally distributed [23]. In this study, a random-effects model was used for data pooling, with Restricted Maximum Likelihood (REML) estimation for variance components. REML is an effective method, particularly for handling heterogeneous datasets, as it provides more accurate estimates of between-study variability. Statistical heterogeneity was assessed using the I^2^ statistic and the Cochran’s Q test (with a significance level of *P* < 0.1). High heterogeneity was considered when I^2^ > 50%, in which case a random-effects model was applied. For studies with I^2^ ≤ 50%, a fixed-effects model was used, and a P-value < 0.05 was considered statistically significant [24, 25]. All analyses were performed using Stata software (version 18.0). Sensitivity analysis was conducted by sequentially excluding each study to assess the stability of the results. Publication bias was evaluated using funnel plots and Egger’s test if more than 10 studies were included in an outcome analysis.

## Results

### Study selection

**Fig. 1:**
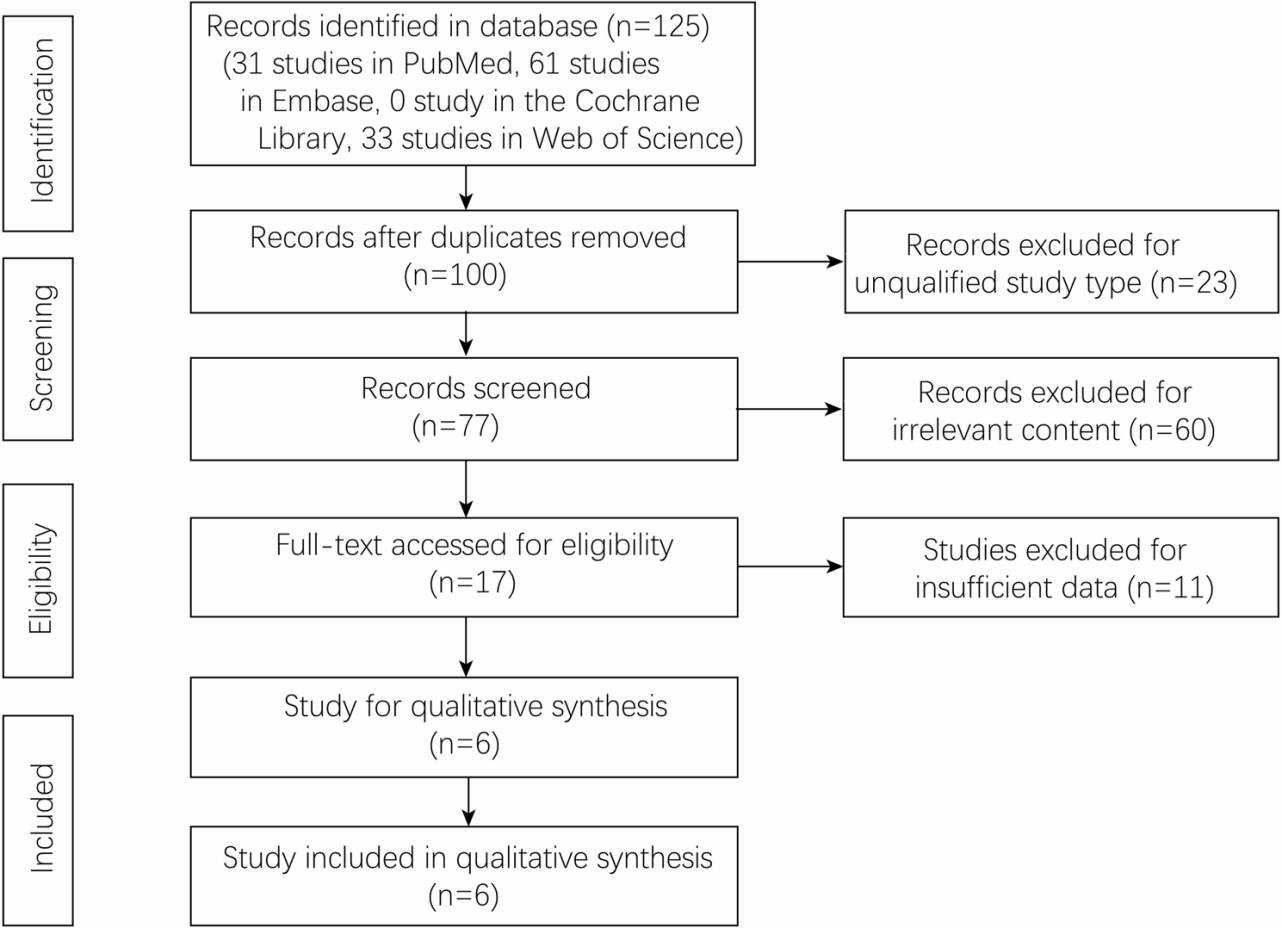
Flowchart of study selection

A total of 125 studies (31 studies in PubMed, 61 studies in Embase, 0 studies in the Cochrane, and 33 studies in Web of Science) were screened in this meta-analysis. There were 100 studies after removing the duplications. The title and abstract were screened by two authors independently, and 17 studies were evaluated at the full-text level. Case reports, reviews, letters, conferences, and comments were excluded. Finally, 6 [8, 18, 26–29] studies that compared PDM group and Non-PDM group after surgery were included in this meta-analysis (Fig. 1).

### Patient characteristics and quality assessment of the included studies

There were 154 patients with PDM and 2283 patients with Non-PDM in the 6 studies. The publication year of the 6 studies were from 2021 to 2023, and all the studies were retrospective studies. Four studies were from Japan, and 2 studies were from China. The grade of complications and the scores of the Newcastle-Ottawa Scale of each study are shown in Table 1.

**Table 1:** Characteristics of the studies included in the meta-analysis abbreviations: PDM persistent descending Mesocolon, NOS Newcastle-Ottawa scale

Author	Year	Country	Study design	Study date	Sample size		Post Operative Complications Clavien-Dindo classification (I/II/III/IV/V)		NOSNOS
					PDM group	Non-PDM	PDM group	Non-PDM	
Hiroaki N	2022	Japan	Retrospective cohort study	2016–2021	12	783	II	II	6
Nitta T	2023	Japan	Retrospective cohort study	2016–2021	13	521	II/III	II/III	6
Mei S	2023	China	Retrospective cohort study	2020–2021	27	105	II/III	II/III	7
Tang G	2022	China	Retrospective cohort study	2018–2022	83	260	II	II/III	7
Hanaoka M	2021	Japan	Retrospective cohort study	2016–2019	10	437	/	/	8
Hamada K	2022	Janan	Retrospective cohort study	2019–2020	9	177	II/Ⅲ/Ⅳ	II/Ⅲ/Ⅳ	6

Abbreviations: *PDM* Persistent Descending Mesocolon, *NOS* Newcastle-Ottawa Scale

### Baseline information

Baseline information, including age, sex, body mass index (BMI), tumor stage, American Society of Anesthesiologists (ASA) score, and location of the tumor. After pooling all the data, the PDM group had a lower tumor stage (OR = 1.70, 95% CI = 1.14 to 2.54, *P* = 0.01) (Table 2). We acknowledge that baseline imbalances in tumor stage may contribute to confounding in our analysis. The retrospective design of the included studies and the lack of adjustment for potential confounders such as tumor stage, surgical approach, and surgeon experience may introduce bias in the pooled results. However, we did not find differences between PDM group and Non-PDM group in age, BMI, sex, location of the cancer, and ASA.

**Table 2:** Summary of characteristics between PDM group and Non-PDM group

Characteristics	Studies	Participants (PDM/Non-PDM)	Mean difference / OR ( 95%CI )	Heterogeneity
Age	5	127/2337	0.14 [-2.99, 3.26]; *P* = 0.93	I^2^ = 42.94%; *P* = 0.15
BMI	5	127/2337	0.74 [-0.41, 1.89]; *P* = 0.21	I^2^ = 30.63%; *P* = 0.34
Male	6	154/2283	1.35 [0.85, 2.16]; *P* = 0.21	I^2^ = 19.90%; *P* = 0.16
Tomor Stage Ⅰ+Ⅱ	3	120/802	1.70 [1.14, 2.54]; *P* = 0.01	I^2^ = 00.00%; *P* = 0.65
Tomor Stage Ⅲ+Ⅳ	3	120/802	0.59 [0.39, 0.88]; *P* = 0.01	I^2^ = 00.00%; *P* = 0.65
Colon Cancer	4	44/1918	0.94 [0.48, 1.85]; *P* = 0.87	I^2^ = 20.29%; *P* = 0.32
Rectal Cancer	4	44/1918	0.93 [0.49, 1.79]; *P* = 0.83	I^2^ = 19.27%; *P* = 0.30
ASA Ⅰ+Ⅱ	3	119/542	1.02 [0.66, 1.59]; *P* = 0.93	I^2^ = 00.00%; *P* = 0.24
ASA Ⅲ	3	119/542	0.98 [0.63, 1.52]; *P* = 0.93	I^2^ = 00.00%; *P* = 0.24

Abbreviations: *PDM* Persistent Descending Mesocolon, *ASA* American Society of Anesthesiologists, *OR* Odd Ratio, *CI* Confidence Interval, *BMI* Body Mass Index

### Operation time

**Fig. 2:**
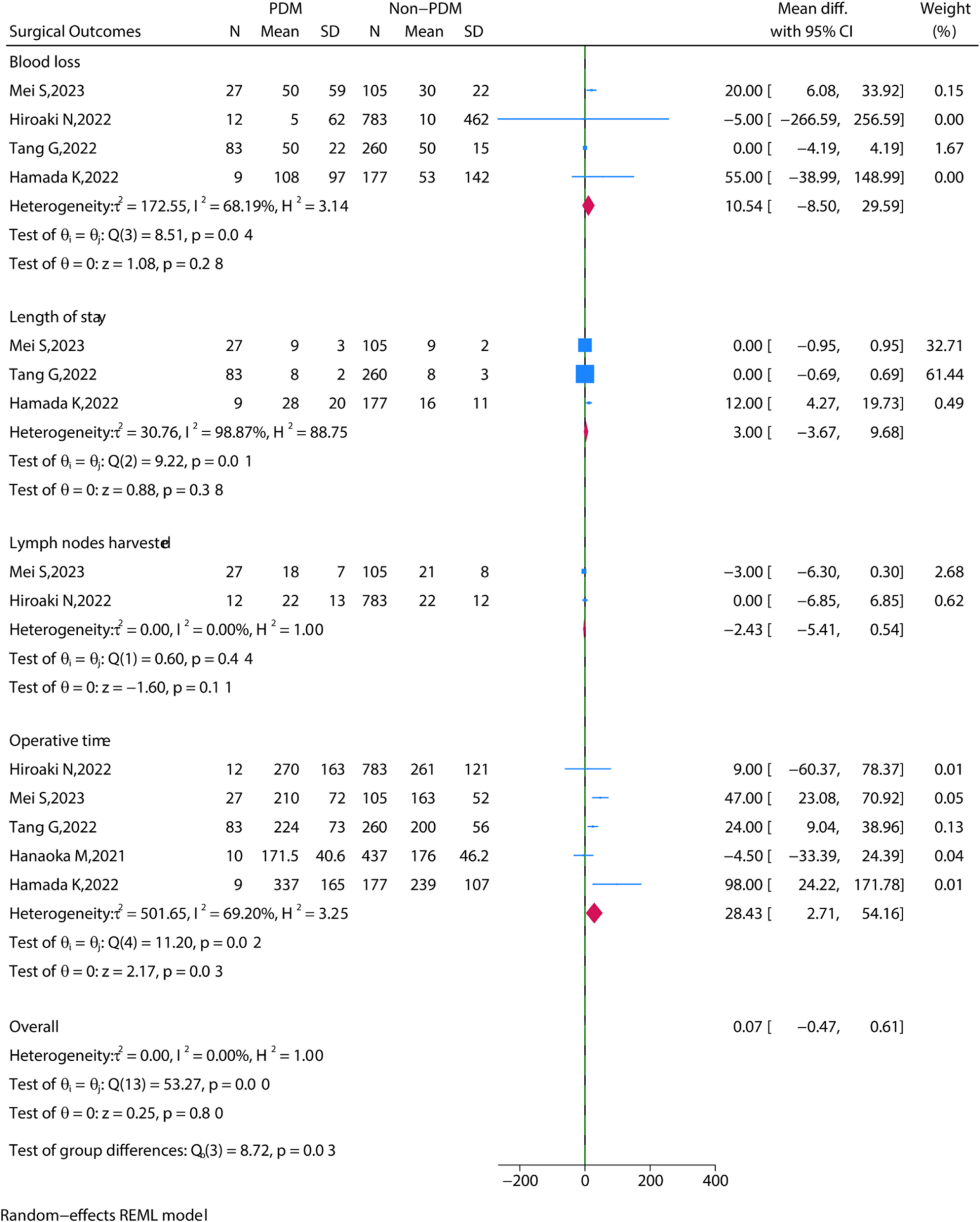
The meta-analysis of operative time

Five studies reported the operation time and the PDM group showed longer operation time than the Non-PDM group (MD = 28.43, 95% CI = 2.71 to 54.16, *P* = 0.03) (Fig. 2).

### Complications

**Fig. 3:**
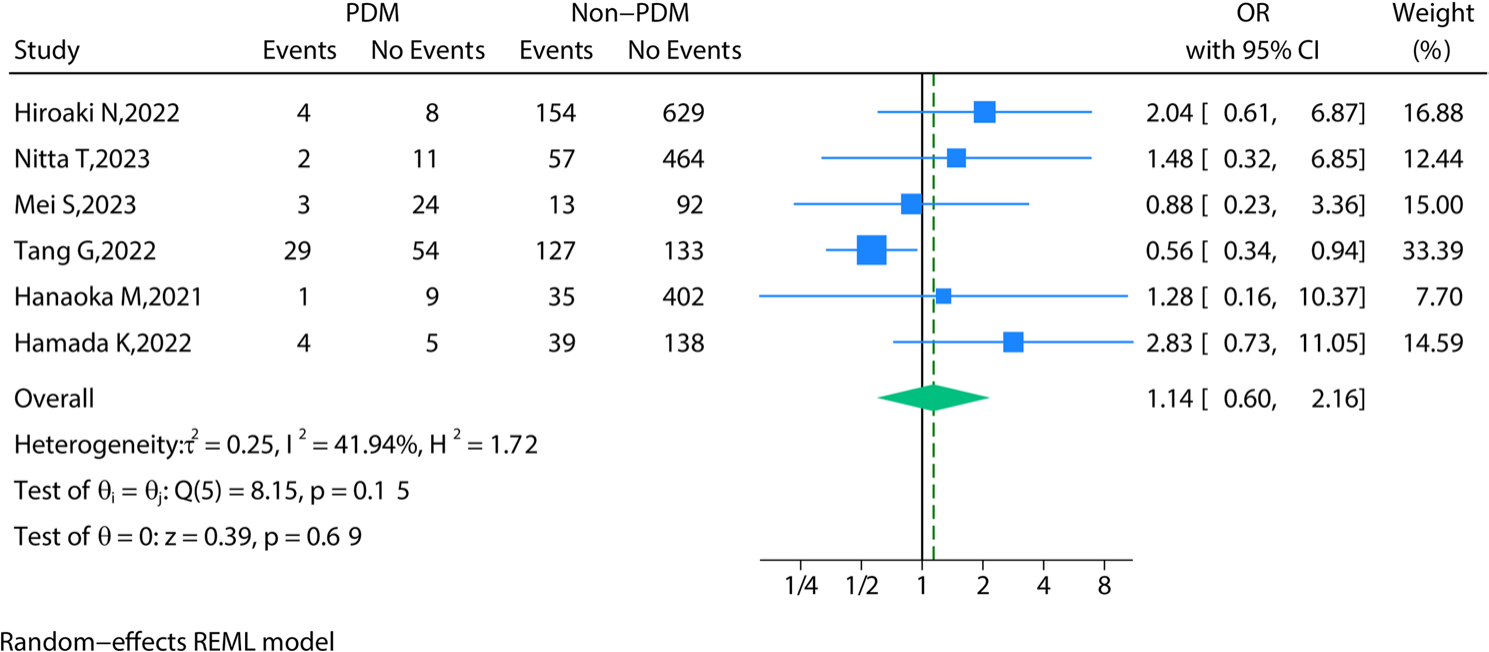
The meta-analysis of overall complications

**Fig. 4:**
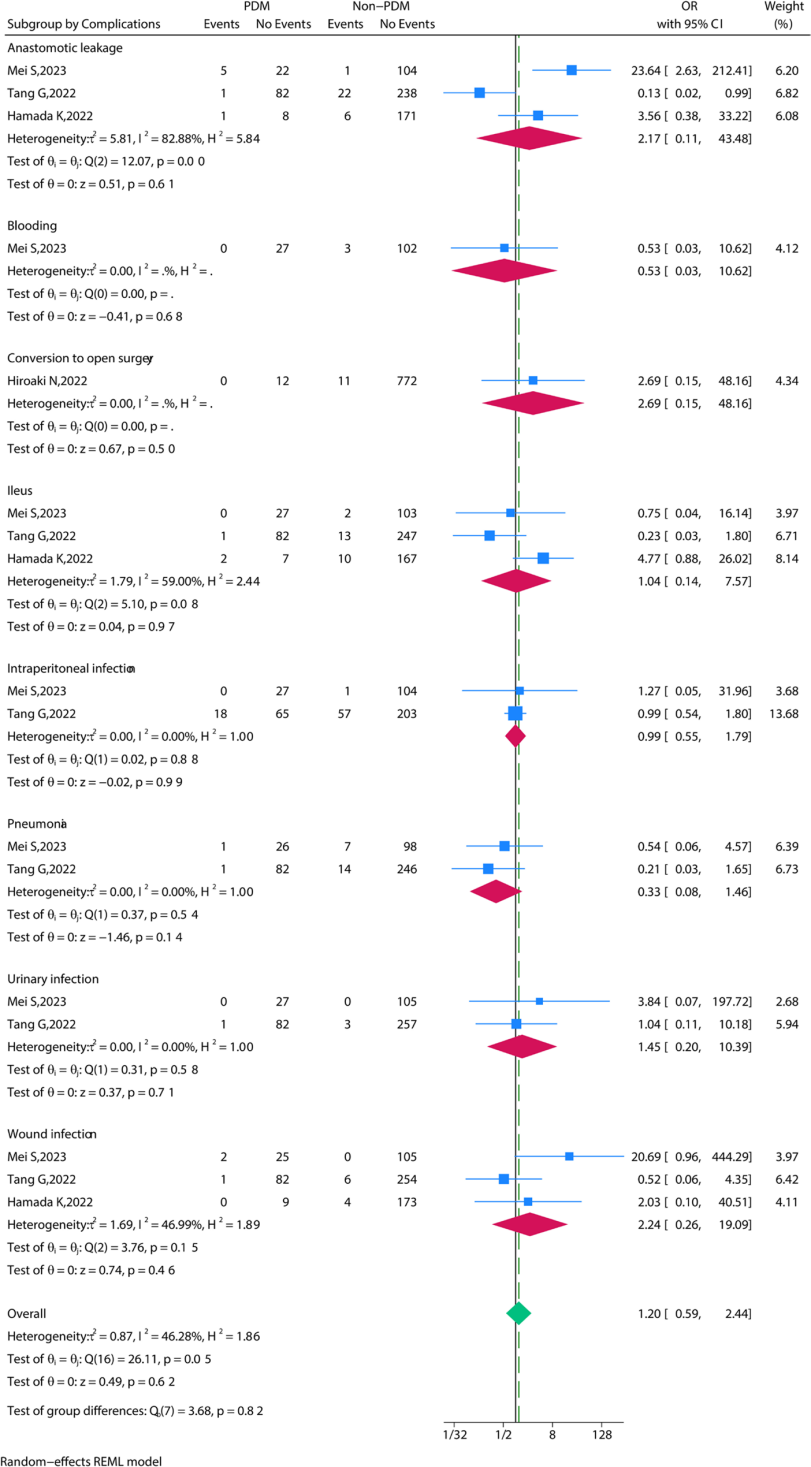
The meta-analysis of different complications

Complications were extracted from 6 studies. Overall complications included, ranging from Clavien-Dindo grade I (mild) to grade V (death). After pooling all the data, no significance in overall complications was found between the PDM group and the Non-PDM group (OR = 1.12, 95% CI = 0.60 to 2.09, *P* = 0.72) (Fig. 3). Moreover, specific complications including anastomotic leakage, intestinal obstruction, abdominal infection, wound infection, bleeding, Pneumonia, Urinary infection, Lymph nodes harvested, and Length of stay were found no statistical significance between the PDM group and the Non-PDM group (Fig. 4). The observed lack of significant differences in postoperative complications between PDM and non-PDM patients may be attributed to the considerable heterogeneity across the included studies. The studies differed in terms of tumor location (rectal vs. colon cancer), metastatic status, tumor stage, and the use of neoadjuvant therapy. These factors are known to influence postoperative recovery and complication rates, and their potential impact should be considered when interpreting the results of this article. Future studies should address these factors through stratified or subgroup analyses to determine how neoadjuvant treatments (chemotherapy or radiochemotherapy), tumor location, and metastatic status may affect postoperative outcomes in patients with PDM. Additionally, more detailed reporting on these factors in primary studies would help clarify their role in the outcomes of PDM patients.

### Sensitivity, consistency, I^2^, and publication bias

**Fig. 5:**
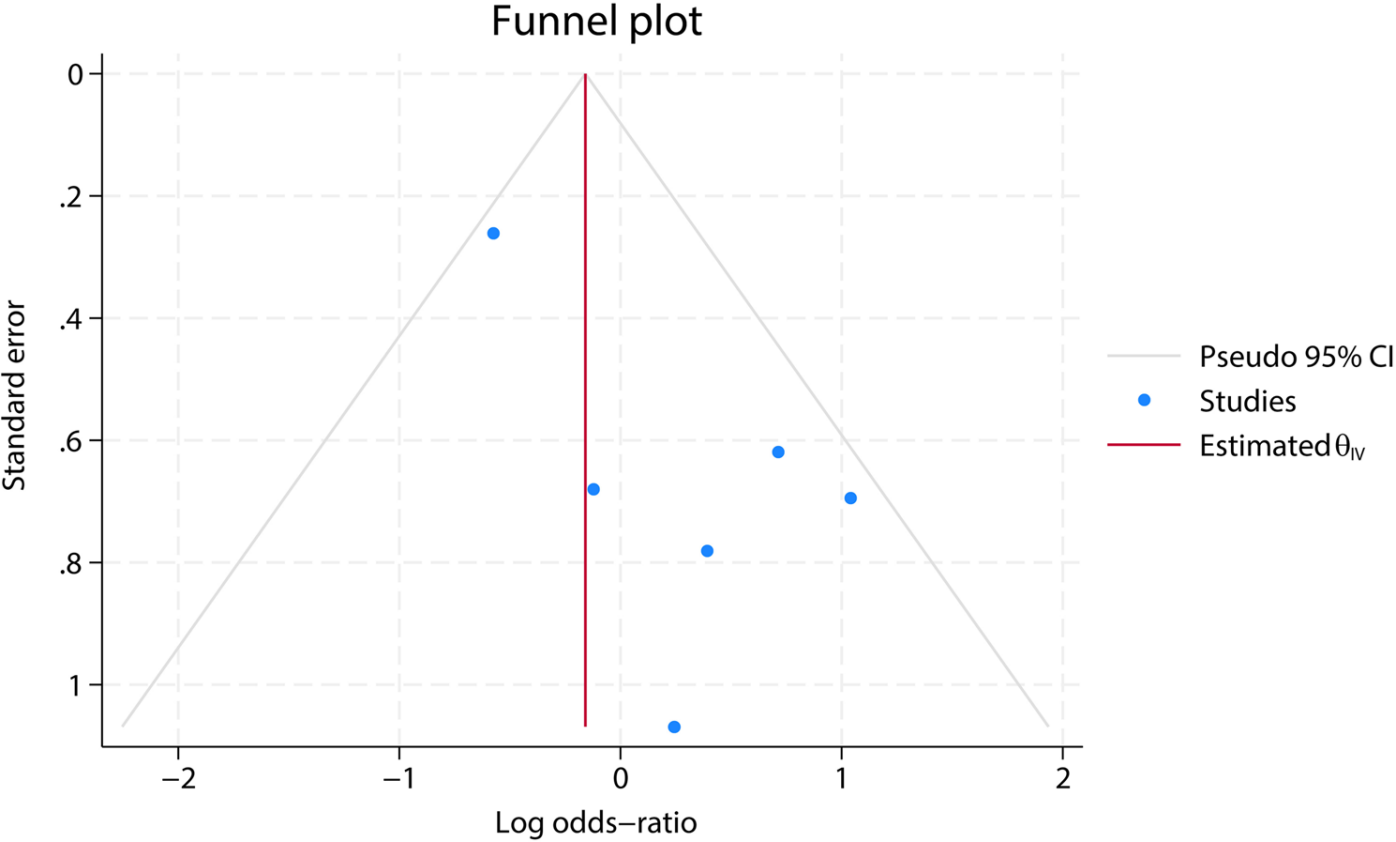
Leave-one-out sensitivity analyses

**Fig. 6:**
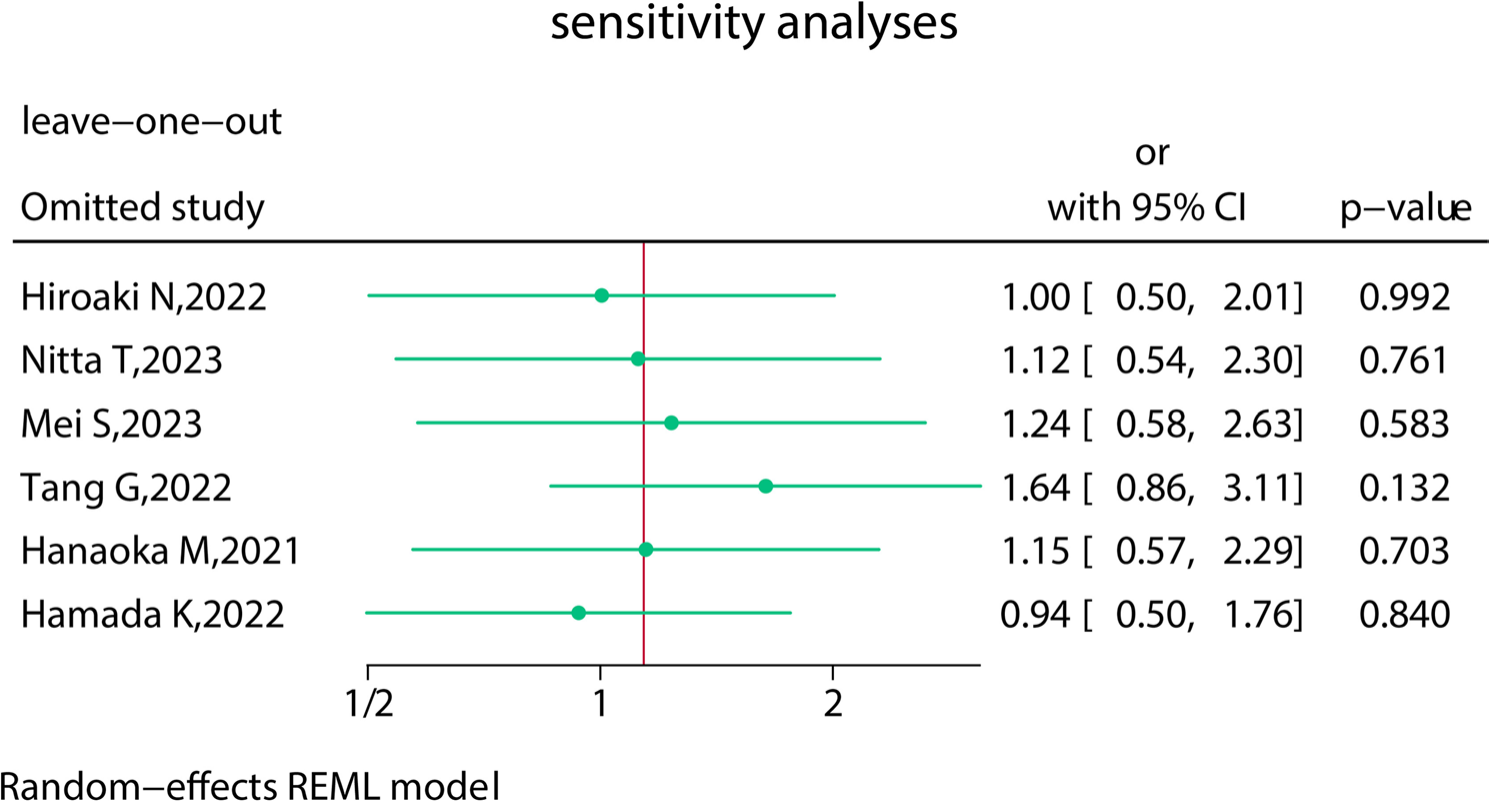
Funnel plot of overall postoperative complications

Repeated meta-analyses were performed by excluding one study in turn as a sensitivity analysis was performed to evaluate the impact of each individual study on the pooled OR and the results were found to be the same (Fig. 5). Consistency was measured by estimating the degree of inconsistency among the results of the studies. Publication bias for the included studies was based on a visual inspection of the funnel plots, which were symmetrical, and no obvious publication bias was found (Fig. 6). However, given the small number of studies included in this meta-analysis, the risk of publication bias cannot be ruled out, and caution is needed in interpreting the findings. The PDM group’s relatively small sample size (*n* = 154) may also contribute to Type II errors, particularly for rare complications like anastomotic leaks and mortality. Therefore, the absence of publication bias should be interpreted cautiously. Furthermore, we performed a qualitative assessment of publication bias by reviewing study registrations and examining whether all pre-specified outcomes were reported. We found although some studies did not fully adhere to their protocols or did not report all intended outcomes, this is common in retrospective studies.

## Discussion

The certainty of evidence for each of the main outcomes was assessed using the GRADE approach. The evidence for postoperative complications was rated as moderate due to the high risk of bias in the included studies and moderate heterogeneity observed in the pooled estimates. Specifically, studies with higher risks of bias in outcome assessment contributed to a moderate rating (Table 3).

**Table 3:** Risk of Bias Summary Table

Author(s)	Selection Bias	Comparability Bias	Outcome Assessment Bias	NOS Score	Risk of Bias
Hiroaki N	High	Low	Low	6/9	High
Hanaoka M	Low	Low	Low	8/9	Low
Mei S	Low	Low	Low	7/9	Low
Nitta T	High	High	Low	6/9	High
Tang G	Low	Low	Low	7/9	Low
Hamada K	High	High	High	6/9	High

For operative time, the evidence was rated as high, as studies consistently reported similar results with low risk of bias and low heterogeneity. This suggests a high level of confidence in the estimated effect of PDM on operative time. The evidence for anastomotic leaks was rated as low because of the substantial risk of bias in the included studies and considerable heterogeneity. Lastly, postoperative hospital stay was rated as moderate because, while results were consistent across studies.

This pooling analysis provides clear evidence that the presence of a Persistent PDM is significantly associated with a prolonged operative time during colorectal surgery. Despite this increased technical complexity, PDM did not lead to a higher incidence of intraoperative or postoperative complications, as no statistically significant differences were observed in any of the surgical outcomes, including anastomotic leak, wound infection, blood loss, or ileus. In other words, while PDM is associated with a statistically significant increase in operative time (MD = 28.43 min), it does not appear to compromise surgical safety or short-term recovery when managed by experienced surgeons. The increased operative time observed in PDM patients should be viewed in the context of the additional surgical complexity it introduces. These findings suggest that PDM patients can still undergo surgery safely, but careful preoperative planning and consideration of surgical techniques are important. To date, no comprehensive synthesis has quantitatively assessed the impact of this anatomical variant on surgical outcomes, underscoring the necessity of this article to inform clinical practice. It is important to note that the PDM group had significantly lower tumor stages compared to the Non-PDM group, which may have contributed to less extensive dissections and easier management. However, despite this advantage, PDM patients still experienced longer operative times, suggesting that PDM introduces its own unique surgical challenges that cannot be fully explained by tumor stage alone. Further studies should explore the interactions between tumor stage and other potential confounding factors, such as surgeon experience, surgical technique, and tumor location.

The consistent and significant elongation of operative time serves as a robust quantitative indicator of the surgical challenge posed by PDM [30, 31]. The aberrant anatomy, the mobile mesocolon and the obliteration of the standard avascular plane of Toldt, necessitates a more meticulous and time-consuming dissection [32–34]. Surgeons must navigate an unfamiliar surgical field with a risk of injuring adjacent structures such as the duodenum, ureter, or mesocolic vessels. Given that, we recommend the use of preoperative CT to identify PDM and aid in preoperative planning. Surgeons should be prepared to modify their surgical technique to accommodate the altered anatomy, particularly in cases where PDM is present. Informed consent discussions should include information about the potential for increased operative time, allowing patients to make fully informed decisions regarding their treatment.

The prolonged operative time observed in PDM patients (MD = 28.43 min) should be understood within the broader context of surgical complexity. Similar to robotic-assisted colorectal surgery, which also increases operative time by 22.7 min compared to laparoscopy [35], PDM extends operative time but does not affect postoperative complications or recovery when managed by experienced surgeons. Additionally, the importance of preoperative anatomical assessment and surgical planning in handling anatomical variations is underscored by Mirza in their studies on surgical complexity [36]. This highlights the need for preoperative imaging to anticipate PDM and adjust surgical techniques, accordingly, ensuring patient safety and reducing operative time. Meanwhile, recent evidence has similarly evaluated perioperative outcomes and complications in colorectal surgery, particularly regarding the use of advanced techniques to assess anastomotic perfusion. Mirza conducted a systematic review and meta-analysis assessing the role of indocyanine green (ICG) fluorescence in reducing anastomotic leaks. Their findings showed that ICG fluorescence significantly reduced clinical anastomotic leaks by approximately 31% compared to conventional methods [37]. This highlights the importance of incorporating technologies like ICG to improve surgical outcomes, especially in cases where surgical complexity is high, such as those involving anatomical variations like PDM. While ICG fluorescence did not significantly impact other perioperative outcomes, such as operative time or hospital stay, its ability to enhance perfusion assessment in high-risk anastomoses makes it particularly valuable in PDM cases, where altered anatomy might affect blood supply. These findings underscore the role of advanced technologies in improving postoperative recovery and reducing complications, even in complex surgical scenarios.

We acknowledge that surgeon experience, institutional surgical volume, and the choice of surgical approach (laparoscopic vs. open surgery) may significantly affect both operative time and postoperative complications, particularly in technically complex procedures such as those involving PDM. These factors were not consistently controlled for across studies, which may have introduced bias and increased variability in the pooled results. Future studies should aim to control for these important confounders to improve the reliability of the findings. The finding that PDM did not increase the risk of complications is critically important, which is an evidence to the adaptability and skill of the operating surgeons [38, 39]. It indicates that the increased intraoperative difficulty, while demanding, can be effectively managed without compromising patient safety or short-term recovery. This finding implies that surgeons, whether through preoperative suspicion on imaging or intraoperative recognition, are effectively identifying and managing this anatomical challenge. This result should reassure clinicians that while PDM makes the operation more demanding, it does not inherently predispose patients to adverse recovery outcomes when managed with appropriate surgical steps.

We observed substantial heterogeneity across the pooled outcomes, particularly for blood loss, anastomotic leakage, and some complication subgroups. We carefully considered potential clinical sources of heterogeneity and conducted qualitative discussion to explore these sources. Tumor location is one of the most likely contributors to heterogeneity in postoperative outcomes, as the anatomical and surgical challenges differ significantly depending on the location of the tumor. Rectal cancer surgeries are generally more complex due to the proximity of the tumor to vital structures such as the bladder, prostate, and pelvic nerves. This complexity often leads to higher rates of anastomotic leakage and increased blood loss, particularly when the tumor is in the lower rectum. Colon cancer, especially in the right colon, typically involves less complicated resections and may explain the lower incidence of complications in these studies. The surgical approach is another key source of heterogeneity. Laparoscopic surgery is widely regarded as having a faster recovery time and fewer complications compared to open surgery due to the smaller incisions and reduced trauma to surrounding tissues. Surgeon experience plays a significant role in surgical outcomes, particularly with newer techniques like robotic-assisted surgery or laparoscopic surgery. Surgeons who are more experienced with these techniques generally achieve better outcomes. All the studies included in this analysis were conducted in East Asia (Japan and China). While this provides important insights into the management of PDM in this region, it limits the generalizability of the findings to Western populations. Surgical techniques, patient demographics, and healthcare infrastructure may differ significantly in other parts of the world. Future studies should include more diverse populations to enhance the external validity of the results.

We observed substantial heterogeneity in some outcomes, such as blood loss and anastomotic leakage. The high I² values indicate significant variability across studies, which could be explained by differences in tumor location, surgical approach, and surgeon experience. Non-significant pooled results with wide confidence intervals should be interpreted with caution.

This study represents the first systematic attempt to pool existing evidence and quantitatively evaluate the impact of PDM on colorectal surgery outcomes. Its strength lies in the rigorous methodology and standardized outcome assessment, which provides the robust estimate to date of the surgical impact of PDM.

Several limitations of this analysis must be acknowledged. First, only six retrospective studies were involved without any cohort studies or randomized controlled trials. Furthermore, the included studies were only from Japan and China, which limits the generalizability of our findings to Western populations. Differences in BMI profiles, surgical infrastructures, and patient demographics may influence the outcomes observed in this study. Future research should aim to include more diverse populations to assess the impact of PDM in different regions and healthcare systems. Future research should include more diverse patient populations to enhance the generalizability of the findings. Lastly, given the relatively small sample size of the PDM group (*n* = 154), particularly for rare postoperative complications such as anastomotic leaks and mortality, there is a potential for Type II errors. This limitation should be considered when interpreting the results, as the study may lack sufficient power to detect significant differences for these rare outcomes.

## Conclusion

No statistically significant differences were observed in complications between PDM and Non-PDM groups, but rather as a key determinant of procedural complexity, quantifiable by a longer operative time. This evidence underscores the importance of preoperative recognition of PDM through careful imaging review. Such awareness will make sure accurate surgical planning, while providing reassurance that the risk of complications could remain comparable to that of a standard procedure. However, it is important to note that the evidence base is observational, and these findings could be influenced by potential biases, including selection bias and unmeasured confounders.

## Supplementary Information

Supplementary Material 1.

Supplementary Material 2.

## Data Availability

The datasets used and analyzed during this study are available upon reasonable request.The datasets used and analyzed during the current study are available from the corresponding author on reasonable request.

## Abbreviations

PDM: Persistent Descending Mesocolon
IQR: Interquartile Range
SD: Standard Deviation
OR: Odds Ratio
CI: Confidence Interval
MD: Mean Difference
REML: Restricted Maximum Likelihood
